# Aldehyde free - Bovine Pericardium – A New Option of Graft in Urethral Stricture Treatment

**DOI:** 10.1590/S1677-5538.IBJU.2024.9928

**Published:** 2024-12-20

**Authors:** Luciano A. Favorito, Rodrigo R. Vieiralves, Arthur V. Batista, Renata Palopoli Silva, Luis Octavio Hauschild, Lucas A. M. Uneda, José A. D. Resende

**Affiliations:** 1 Hospital Federal da Lagoa Serviço de Urologia Rio de Janeiro RJ Brasil Serviço de Urologia – Hospital Federal da Lagoa, Rio de Janeiro, RJ, Brasil

## Abstract

**Objective:**

The current management for complex urethral strictures commonly uses open reconstruction with buccal mucosa urethroplasty. However, there are multiple situations whereby buccal mucosa is inadequate (pan-urethral stricture or prior buccal harvest) or inappropriate for utilization (heavy tobacco use or oral radiation). Multiple options exist for use as alternatives or adjuncts to buccal mucosa in complex urethral strictures (injectable antifibrotic agents, augmentation urethroplasty with skin flaps, lingual mucosa, bladder mucosa, colonic mucosa, and new developments in tissue engineering for urethral graft material) (1, 2). In the present video, we present a case where we used a new option of graft to treat urethral strictures: the L-Hydro® tissue treatment technology 100% aldehyde free, VIVENDI graft.

**Materials and Methods:**

The present study was approved according to the ethical standards of the hospital's institutional committee on experimentation with human beings. A 57 year-old male patient developed a urethral stricture due to prolonged use of a urinary catheter during a previous hospitalization. A cystourethrogram was performed, which revealed a stenosis of the penile urethra measuring 2.5 cm in length. Urethroplasty was proposed for the surgical treatment in this case. We used a longitudinal penile incision with a ventral sagittal urethrotomy in the penile stricture. A free VIVENDI graft was placed into the longitudinal incision in the dorsal urethra and fixed with interrupted suture as dorsal inlay. The ventral urethrotomy was closed over a 16Fr Foley catheter and the skin incision was then closed in layers. The patient will receive post-operative follow-up for 3 months for clinical assessment through symptoms, uroflowmetry, urethroscopy and residual urine volume after urination.

**Results:**

No intraoperative or postoperative complications occurred. The patient could achieve satisfactory voiding and no complication was seen during the three-month follow-up. Four weeks after surgery, he underwent urethroscopy, which revealed a good appearance of the urethra, with no stenosis or signs of infection.

**Conclusion:**

In the present case the use of bovine pericardium graft for the treatment of penile urethral stricture had a good result and can be an option to repair complex urethral strictures. However, the results presented require a larger population group in addition to multicenter studies with longer follow-up time to ensure the findings obtained.

Available at: http://www.intbrazjurol.com.br/video-section/20249928_Vieiralves_et_al
